# Regulation of 5‐fluorodeoxyuridine monophosphate‐thymidylate synthase ternary complex levels by autophagy confers resistance to 5‐fluorouracil

**DOI:** 10.1096/fba.2022-00099

**Published:** 2022-11-11

**Authors:** Nana Nishizawa, Chinatsu Kurasaka, Yoko Ogino, Akira Sato

**Affiliations:** ^1^ Department of Biochemistry and Molecular Biology, Faculty of Pharmaceutical Sciences Tokyo University of Science Chiba Japan; ^2^ Present address: Kowa Company Ltd. Nihonbashi‐Honcho Tokyo Japan; ^3^ Present address: Department of Gene Regulation, Faculty of Pharmaceutical Sciences Tokyo University of Science Chiba Japan

**Keywords:** 5‐fluorodeoxyuridylate covalent complex with thymidylate synthase, 5‐fluorouracil, autophagy, colorectal cancer, drug resistance, thymidylate synthase, 5‐fluorodeoxyuridylate

## Abstract

5‐Fluorouracil (5‐FU) is a cornerstone drug used to treat colorectal cancer (CRC). However, the prolonged exposure of CRC cells to 5‐FU results in acquired resistance. We have previously demonstrated that levels of the 5‐fluorodeoxyuridylate (FdUMP) covalent complex with thymidylate synthase (FdUMP‐TS) and free‐TS (native enzyme) are higher in 5‐FU‐resistant CRC cells than in the parental cell line (HCT116). Accordingly, resistant cells may have an efficient system for trapping and removing FdUMP‐TS, thus imparting resistance. In this study, using a model of 5‐FU‐resistant CRC cells generated by repeated exposure, the role of autophagy in the elimination of FdUMP‐TS in resistant cells was investigated. The resistant cells showed greater sensitivity to autophagy inhibitors than that of parental cells. Autophagy inhibition increased 5‐FU cytotoxicity more substantially in resistant cells than in parental cells. Furthermore, autophagy inhibition increased FdUMP‐TS protein accumulation in resistant cells. Our findings suggest that resistance to 5‐FU is mediated by autophagy as a system to eliminate FdUMP‐TS and may guide the use and optimization of combination therapies involving autophagy inhibitors.

## Graphical abstract

The 5‐FU‐resistant HCT116 cells showed greater sensitivity to autophagy inhibitors than that of parental cells. In addition, autophagy inhibition increased 5‐FU cytotoxicity more substantially in resistant cells than in parental cells. Furthermore, autophagy inhibition increased FdUMP‐TS protein accumulation in resistant cells.

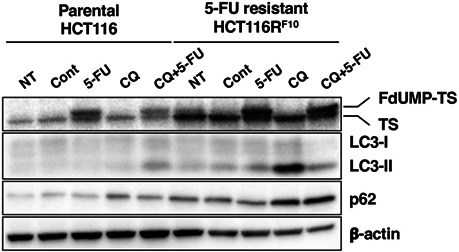

## 1 INTRODUCTION

The properties of 5‐fluorouracil (5‐FU), including its chemical synthesis and widespread anticancer effects, in fundamental and clinical terms were first published in 1957.^1^, ^2^, ^3^ 5‐FU is still widely used today, mainly for the treatment of gastrointestinal cancers, such as colorectal cancer (CRC).^4^, ^5^ 5‐FU is converted to the active metabolite 5‐fluorodeoxyuridine monophosphate (FdUMP), which is a potent inhibitor of thymidylate synthase (TS).^3^, ^5^, ^6^, ^7^, ^8^ FdUMP forms a ternary complex with TS and 5, 10‐methylenetetrahydrofolate (5, 10‐CH_2_‐THF).^4^, ^5^, ^6^, ^7^, ^8^, ^9^ TS catalyzes the conversion of dUMP to dTMP using the co‐enzyme 5, 10‐CH_2_‐THF as a methyl donor.^10^ The ternary complex inhibits TS function, depletes intracellular dTTP, dNTP pools, and subsequently inhibits DNA synthesis.^3^, ^4^, ^5^, ^8^ In addition, 5‐FU can exert anticancer effects through its incorporation into DNA and RNA as the active metabolites fluorodeoxyuridine triphosphate (FdUTP) and fluorouridine triphosphate (FUTP), respectively.^3^, ^4^, ^5^

The mechanisms underlying resistance to 5‐FU and its derivatives have been studied extensively,^4^, ^5^, ^11^ including studies of the function and/or expression of TS and other enzymes related to the 5‐FU anabolism and catabolism nucleic acid metabolism pathway.^4^, ^5^, ^11^ In particular, TS contributes to increased 5‐FU sensitivity and it is thought that targeting TS is an excellent strategy to reverse 5‐FU resistance.^4^, ^5^, ^11^, ^12^ Many studies have shown that the amplification of *TYMS*, leading to *TYMS* mRNA overexpression and TS protein overproduction, is a major mechanism underlying resistance to 5‐FU and its derivatives.^12^, ^13^, ^14^, ^15^ Interestingly, the binding of the TS protein to its own mRNA leads to the formation of an autoregulatory feedback loop that represses the translation of *TYMS* mRNA.^16^, ^17^, ^18^, ^19^ One important 5‐FU resistance mechanism is the disruption of the autoregulatory feedback loop for the repression of translation.^18^, ^19^, ^20^ TS ligands, such as the 5‐FU active metabolites FdUMP and dUMP, disrupt the binding of the TS enzyme to *TYMS* mRNA, leading to translational derepression and overproduction of the TS enzyme.^18^, ^19^, ^20^ In addition, it has been proposed that fluoropyrimidine increases TS levels via its effect on TS enzyme stability, with no effect on *TYMS* mRNA.^21^, ^22^, ^23^ These observations indicated that detailed analyses of translational derepression, enzyme stabilization, and gene amplification in the process of TS induction can help to elucidate the mechanism underlying the acquisition of 5‐FU resistance.

Furthermore, we have shown that 5‐FU resistant CRC cells show higher TYMS expression relative to that in 5‐FU‐sensitive parental CRC cells and use a fraction of TS to trap FdUMP, which results in resistance to 5‐FU and its derivatives.^24^ We have recently shown that the trapping of FdUMP by TS is more effective in 5‐FU‐resistant CRC cells than in parental CRC cells.^24^, ^25^ We predict that the regulation of the balance between the storage of the active free TS form and the accumulation of the FdUMP‐TS‐5, 10‐CH_2_‐THF covalent complex is responsible for direct resistance to 5‐FU.^25^ In addition, other known mechanisms underlying 5‐FU resistance include the perturbance of cell death, autophagy, altered epigenetic repression, and expression/functional changes in drug transporters and noncoding RNA.^4^, ^5^, ^11^ Among these, autophagy has an important role in drug sensitivity and resistance to cancer chemotherapy with 5‐FU.^5^, ^26^ The inhibition of autophagy potentiates the cytotoxic effect of 5‐FU in CRC cells.^27^, ^28^, ^29^ The combination of 5‐FU and autophagy inhibitors, 3‐methyladenine (3‐MA) or chloroquine (CQ), inhibits 5‐FU‐induced autophagy and enhances 5‐FU induced tumor growth inhibition.^28^, ^30^

In this study, we investigated the involvement of autophagy in the accumulation and elimination of a FdUMP‐TS in 5‐FU‐resistant CRC HCT116R^F10^ cells. We evaluated the effects of the autophagy inhibitors CQ and bafilomycin A1 (BafA1) on the sensitivity of HCT116R^F10^ cells and parental HCT116 cells to 5‐FU and the accumulation of FdUMP‐TS. We discuss the possibility of FdUMP trapping by the TS enzyme and elimination of the FdUMP‐TS enzyme as a key mechanism underlying 5‐FU resistance.

## 2 MATERIALS AND METHODS

### 2.1 Reagents

5‐FU was obtained from FUJIFILM Wako Pure Chemical. 5‐FU was stored as 100 mM stock solutions in dimethyl sulfoxide (DMSO, Sigma‐Aldrich; Merck KGaA, Darmstadt, Germany) at −20°C. The autophagy inhibitors CQ and BafA1 were bought from Sigma‐Aldrich and LKT Laboratories, respectively. CQ and BafA1 were stored at 60 mM in water and 0.8 mM in DMSO at −20°C, respectively.

### 2.2 Cells and culture conditions

The human CRC cell line HCT116 was obtained from the American Type Culture Collection (Manassas, VA, USA). 5‐FU‐resistant HCT116 (HCT116R^F10^) cells were developed according to a previously described method.^24^, ^25^ Parental HCT116 and 5‐FU‐resistant HCT116R^F10^ cell lines were then cultured as described previously.^24^, ^25^ Both parental HCT116 cells and 5‐FU‐resistant HCT116R^F10^ cells were grown in Dulbecco's Modified Eagle Medium (D‐MEM, Cat# 043–30085; FUJIFILM Wako Pure Chemical). [Correction added on [7^th^ December 2022], after first online publication: Cat# corrected from “043‐30,085 to 043‐30085.] The culture media contained 10% heat‐inactivated fetal bovine serum, 100 units/ml penicillin, and 100 μg/ml streptomycin.

### 2.3 Colony formation assay

A colony formation assay was performed as described previously.^24^, ^25^ Cells were dissociated with Accutase, suspended in medium, inoculated into 6‐well plates (200 cells per well) in duplicate, and incubated overnight. The cells were treated with various concentrations of drugs or with solvent (DMSO or water) as a negative control. After a 10‐day incubation, the cells were fixed with 4% formaldehyde solution and stained with 0.1% (w/v) crystal violet. Colonies in each well were counted. The sensitivity index (SI) was defined as the ratio of EC_50_ values between the resistant and parental cell lines.

### 2.4 Western blotting

Western blotting was performed as described previously.^24^, ^25^ The antibodies used were rabbit anti‐thymidylate synthase (D5B3) monoclonal antibody (9045 S; 1:1000, Cell Signaling Technologies), rabbit anti‐LC3 polyclonal antibody (PM036, 1:1000; Medical & Biological Laboratories), mouse anti‐SQSTM1(D‐3) monoclonal antibody (sc‐28359, 1:200; Santa Cruz Biotechnology), mouse anti‐beta‐actin monoclonal antibody (A19178‐200UL, 1:20,000; Sigma‐Aldrich), horseradish peroxidase‐linked anti‐rabbit IgG (1:20,000; GE Healthcare), and horseradish peroxidase‐linked whole antibody anti‐mouse IgG (1:20,000; GE Healthcare). [Correction added on [7^th^ December 2022], after first online publication: sc‐28,358 has been corrected to sc‐28359.]

### 2.5 Statistical analysis

Statistical analyses were performed using GraphPad Prism 9. The data are presented as means ± standard error (SE). Significant differences among groups were evaluated using Student's *t*‐tests and one‐way analysis of variance. A value of *p* < 0.05 was considered significant.

## 3 RESULTS

### 3.1 Autophagy inhibitors are more effective in 5‐FU‐resistant HCT116R^F10^ cells than in parental HCT116 cells

**FIGURE 1:**
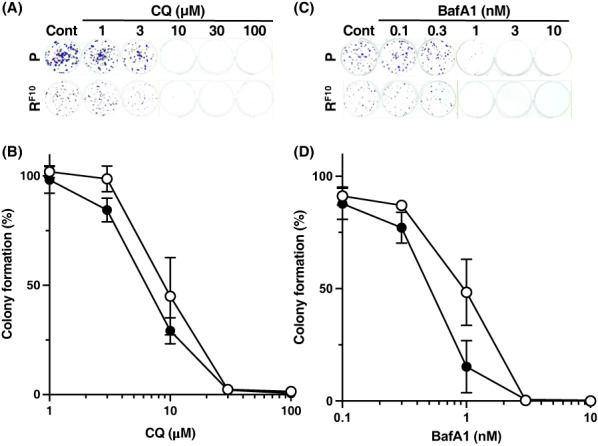
Cytotoxic effects of autophagy inhibitors, chloroquine and bafilomycin A1, in 5‐FU‐resistant HCT116R^F10^ cells and parent HCT116 cells. (A) Image of colony formation by HCT116R^F10^ cells and HCT116 cells after treatment with chloroquine (CQ). (B) Sensitivity of CQ in HCT116R^F10^ cells and HCT116 cells determined by a colony formation assay. Cells were treated with the indicated concentration of CQ and incubated for 10 days. Colony formation (%) represents the average of three independent experiments, with error bars indicating the SE of triplicates. Solid circle, HCT116R^F10^ cells; open circle, HCT116 cells. (C) Image of colony formation in HCT116R^F10^ cells and HCT116 cells after treatment with bafilomycin A1 (BafA1). (D) Cells were treated with the indicated concentration of BafA1 and incubated for 10 days. Colony formation (%) is reported as the average of three independent experiments, with error bars showing the SE of triplicates. Solid circle, HCT116R^F10^ cells; open circle, HCT116 cells. [Correction added on [7^th^ December 2022], after first online publication: “concentration of CQ > BafA1” has been corrected to: “concentration of BafA1”.]

We investigated the association between autophagy and 5‐FU resistance in our established human CRC 5‐FU‐resistant HCT116R^F10^ cells and parental HCT116 cells. The sensitivities to the autophagy inhibitors CQ and BafA1 in HCT116 and HCT116R^F10^ cells were examined by a colony formation assay (Figure 1). The CQ inhibits autophagy flux by decreasing autophagosome‐lysosome fusion.^31^ The BafA1 suppresses autophagy flux by inhibiting both V‐ATPase dependent acidification and autophagosome‐lysosome fusion.^32^ In addition, the 50% effective concentrations (EC_50_) of both autophagy inhibitors in parental HCT116 and HCT116R^F10^ cells were determined by a colony formation assay (Table 1). As shown in Table 1, Figure 1A,B, HCT116R^F10^ cells had 1.6‐fold (EC_50_ = 6.4 μM) higher sensitivity to CQ than that of the parental HCT116 cells (EC_50_ = 10 μM). Similarly, the EC_50_ values for BafA1 were 1.8‐fold lower in HCT116R^F10^ cells (EC_50_ = 0.54 nM) than in HCT116 cells (EC_50_ = 0.95 nM) (Table 1, Figure 1C,D). These data indicate that 5‐FU‐resistant HCT116R^F10^ cells were more sensitive to CQ and BafA1 compared to parental HCT116 cells. In addition, these findings show that autophagy is actively involved in the survival and proliferation of 5‐FU‐resistant CRC HCT116R^F10^ cells.

**TABLE 1:** Sensitivity to autophagy inhibitors in parental HCT116 cells and 5‐FU‐ resistant HCT116R^F10^ cells

Inhibitor	EC_50_		SI
	HCT116	HCT116R^F10^	
Chloroquine	10.0 ± 3.5 μM	6.4 ± 1.2 μM	1.6
Bafilomycin A1	1.0 ± 0.4 nM	0.5 ± 0.2 nM	2.0

*Note*: EC_50_ values are presented as mean ± SD.

Abbreviations: EC_50_, 50% effective concentration; SI, sensitivity index.

### 3.2 Inhibition of autophagy enhances the anticancer effect of 5‐FU on 5‐FU‐resistant HCT116R^F10^ cells

**FIGURE 2:**
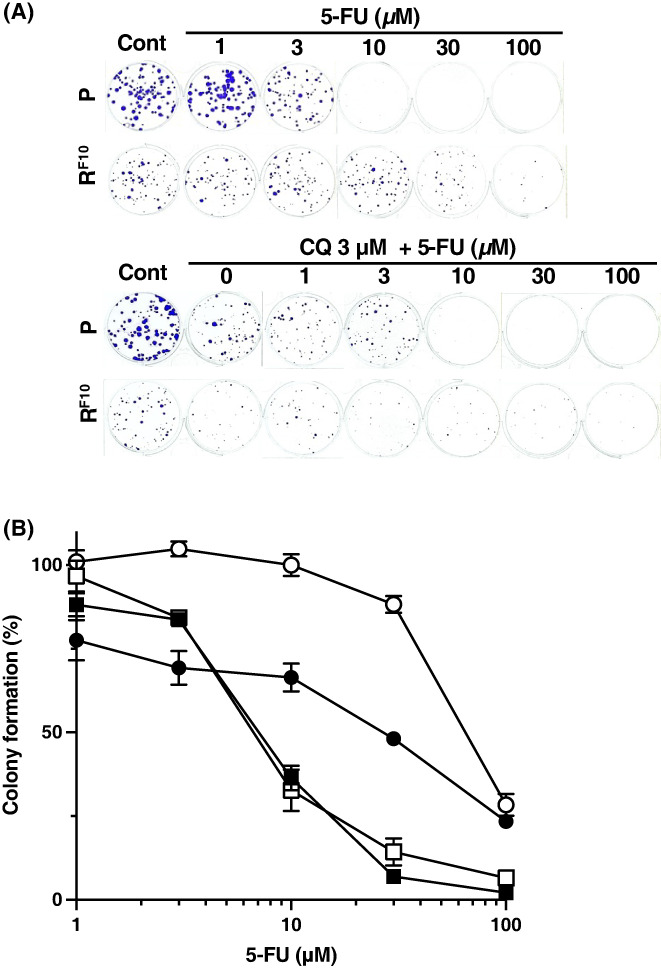
Anticancer effect of the combination of 5‐FU and the autophagy inhibitor chloroquine in 5‐FU resistant HCT116R^F10^ cells and parent HCT116 cells. (A) Image of colony formation in HCT116R^F10^ cells and HCT116 cells after co‐treatment with chloroquine (CQ) and 5‐FU. (B) Anticancer effect of the combination of CQ and 5‐FU in HCT116R^F10^ cells and HCT116 cells determined by a colony formation assay. Cells were treated with 3 μM CQ and the indicated concentration of 5‐FU, followed by incubation for 10 days. Colony formation (%) is presented as the average of three or six independent experiments, with error bars showing the SE of triplicates. Solid circle, combination of CQ and 5‐FU in HCT116R^F10^ cells; open circle, treatment with 5‐FU alone in HCT116R^F10^ cells; Solid square, combination of CQ and 5‐FU in HCT116 cells; open square, treatment with 5‐FU alone in HCT116 cells. Colony formation (%) in 3, 10 and 30 μM 5‐FU indicates the mean of six independent experiments. [Correction added on [7^th^ December 2022], after first online publication: “Solid square, combination of CQ and 5‐FU in HCT116R^F10^ cells; open square, treatment with 5‐FU alone in HCT116 cells. # indicates > Colony formation (%) in 3, 10 and 30 μM 5‐FU indicate” has been corrected to “Solid square, combination of CQ and 5‐FU in HCT116 cells; open square, treatment with 5‐FU alone in HCT116 cells. Colony formation (%) in 3, 10 and 30 μM 5‐FU indicates the mean of six independent experiments”.]

To explore the mechanism by which autophagy conferred resistance to 5‐FU, we analyzed the sensitivity to 5‐FU in HCT116R^F10^ cells and parental HCT116 cells after co‐treatment with the autophagy inhibitors CQ or BafA1. [Correction added on [7^th^ December 2022], after first online publication: “sensitivity of HCT116R_F10_ cells” has been corrected to: “sensitivity to 5‐FU in HCT116R_F10_ cells”.] Interestingly, HCT116R^F10^ cells showed higher sensitivity to 5‐FU when co‐treated with CQ than when treated with 5‐FU alone. In contrast, the sensitivity of 5‐FU in parental HCT116 cells was unaffected by CQ co‐treatment (Figure 2A,B). The EC_50_ values of 5‐FU in parental HCT116 cells and HCT116R^F10^ cells with or without CQ co‐treatment were determined by a colony formation assay (Table 2). The EC_50_ of 5‐FU in HCT116R^F10^ cells was higher for 5‐FU alone (EC_50_ = 64.7 μM) than for co‐treatment with CQ (EC_50_ = 25.8 μM). In contrast, the EC_50_ of 5‐FU in parental HCT116 cells was lower for 5‐FU alone (EC_50_ = 6.8 μM) than for co‐treatment with CQ (EC_50_ = 7.3 μM) (Table 2, Figure 2A,B). Regarding the sensitivity index of 5‐FU, HCT116R^F10^ cells were 2.5‐fold more sensitive to 5‐FU when co‐treated with CQ. In contrast, HCT116 cells showed similar sensitivity to 5‐FU with and without CQ co‐treatment. These findings indicate that the combination of 5‐FU and CQ enhanced the cell growth inhibition and cytotoxic effects of 5‐FU in HCT116R^F10^ cells.

**TABLE 2:** Anticancer sensitivities of 5‐FU in the parental HCT116 cells and 5‐FU‐resistant HCT116R^F10^ cells with or without autophagic inhibitors, chloroquine and bafilomycin A1

Cell lines	EC_50_ (5‐FU, μM)			SI	
	5‐FU alone	+ CQ	+ BafA1	+ CQ	+ BafA1
HCT116	6.8 ± 1.1	7.3 ± 1.1	4.4 ± 0.3	0.9	1.5
HCT116R^F10^	64.7 ± 5.0	25.8 ± 3.5	37.3 ± 23.5	2.5	1.7

*Note*: EC_50_ values indicate mean ± SD.

Abbreviations: BafA1, bafilomycin A1; CQ, chloroquine; EC_50_, 50% effective concentration; SI, sensitivity index. [Correction added on [7^th^ December 2022], after first online publication: Table title corrected from “Anticancer sensitivities of 5‐FU in the parental HCT116 cells and 5‐FU‐resistant HCT116R^F10^ cells with or without autophagic inhibitors, chloroquine and bafilomycin A > A1” has been corrected to “Anticancer sensitivities of 5‐FU in the parental HCT116 cells and 5‐FU‐resistant HCT116R^F10^ cells with or without autophagic inhibitors, chloroquine and bafilomycin A1” and fourth column heading corrected from “+ Baf A1” to “+ BafA1”.]

**FIGURE 3:**
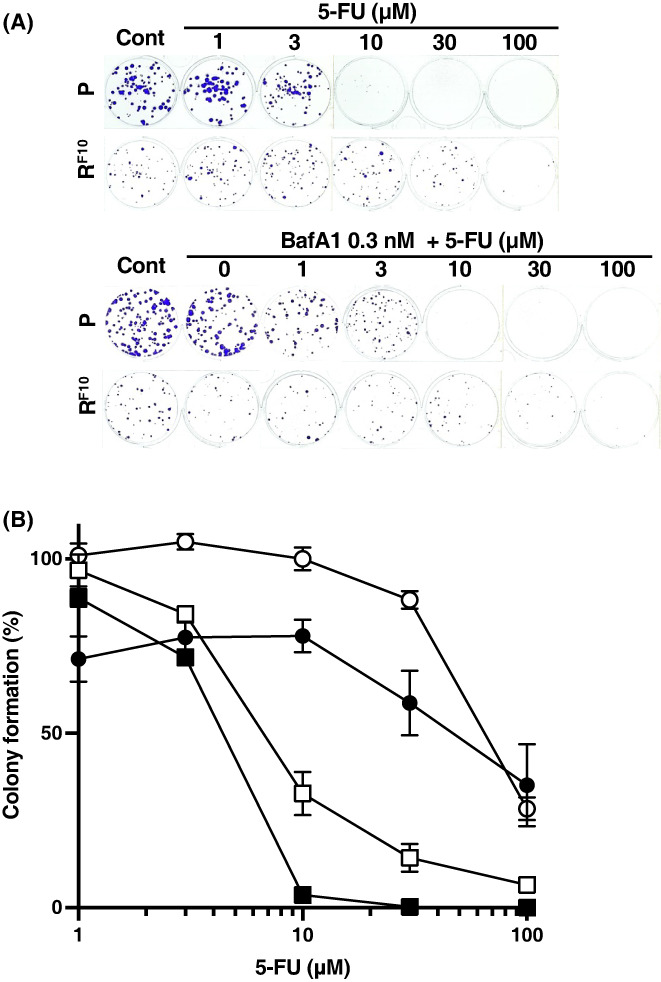
Anticancer effect of the combination of 5‐FU and the autophagy inhibitor bafilomycin A1 in 5‐FU‐resistant HCT116R^F10^ cells and parent HCT116 cells. (A) Image of colony formation in HCT116R^F10^ cells and HCT116 cells after co‐treatment with bafilomycin A1 (BafA1) and 5‐FU. (B) Anticancer effect of the combination of BafA1 and 5‐FU in HCT116R^F10^ cells and HCT116 cells determined by a colony formation assay. Cells were treated with 0.3 nM BafA1 and the indicated concentration of 5‐FU, followed by incubation for 10 days. Colony formation (%) is presented as the average of three or six independent experiments, with error bars showing the SE of triplicates. Solid circle, combination of BafA1 and 5‐FU in HCT116R^F10^ cells; open circle, treatment with 5‐FU alone in HCT116R^F10^ cells; Solid square, combination of BafA1 and 5‐FU in HCT116 cells; open square, treatment with 5‐FU alone in HCT116 cells. Colony formation (%) in 3, 10 and 30 μM 5‐FU indicates the mean of six independent experiments. [Correction added on [7^th^ December 2022], after first online publication: “Solid square, combination of Baf1 and 5‐FU in HCT116R^F10^ cells; open square, treatment with 5‐FU alone in HCT116 cells. # indicates > Colony formation (%) in 3, 10 and 30 μM 5‐FU indicate” has been corrected to “Solid square, combination of BafA1 and 5‐FU in HCT116 cells; open square, treatment with 5‐FU alone in HCT116 cells. Colony formation (%) in 3, 10 and 30 μM 5‐FU indicates the mean of six independent experiments.”]

Similarly, the anticancer effect in HCT116R^F10^ cells was higher for co‐treatment with 5‐FU and BafA1 than with 5‐FU alone (Figure 3A,B and Figure S1). The EC_50_ of 5‐FU in HCT116R^F10^ cells was higher for 5‐FU alone (EC_50_ = 64.7 μM) than for co‐treatment with Baf1 (EC_50_ = 37.3 μM). On the other hand, the EC_50_ of 5‐FU in parental HCT116 cells was slightly higher for 5‐FU alone (EC_50_ = 6.8 μM) than for co‐treatment with BafA1 (EC_50_ = 4.4 μM) (Table 2, Figure 3A,B). Of note, the anticancer effect of 5‐FU in parental HCT116 cells was slightly higher with co‐treatment with BafA1 than for 5‐FU alone (Figure 3A,B and Figure S1). As shown in Figure S1, HCT116 cells and HCT116R^F10^ cells were treated with the indicated concentrations of 5‐FU (approximately EC_20_ values: 3 μM for HCT116 cells; 30 μM for HCT116R^F10^ cells), respectively. These results suggest that the inhibition of autophagy had a greater influence on the anticancer effects of 5‐FU on HCT116R^F10^ cells than on HCT116 cells. Our findings suggested that the autophagy machinery regulates 5‐FU resistance in HCT116R^F10^ cells.

### 3.3 Inhibition of autophagy prevents the removal of the ternary complex thymidylate synthase with 5‐fluorodeoxyuridylate in 5‐FU‐resistant HCT116R^F10^ cells compared to parental HCT116 cells

**FIGURE 4:**
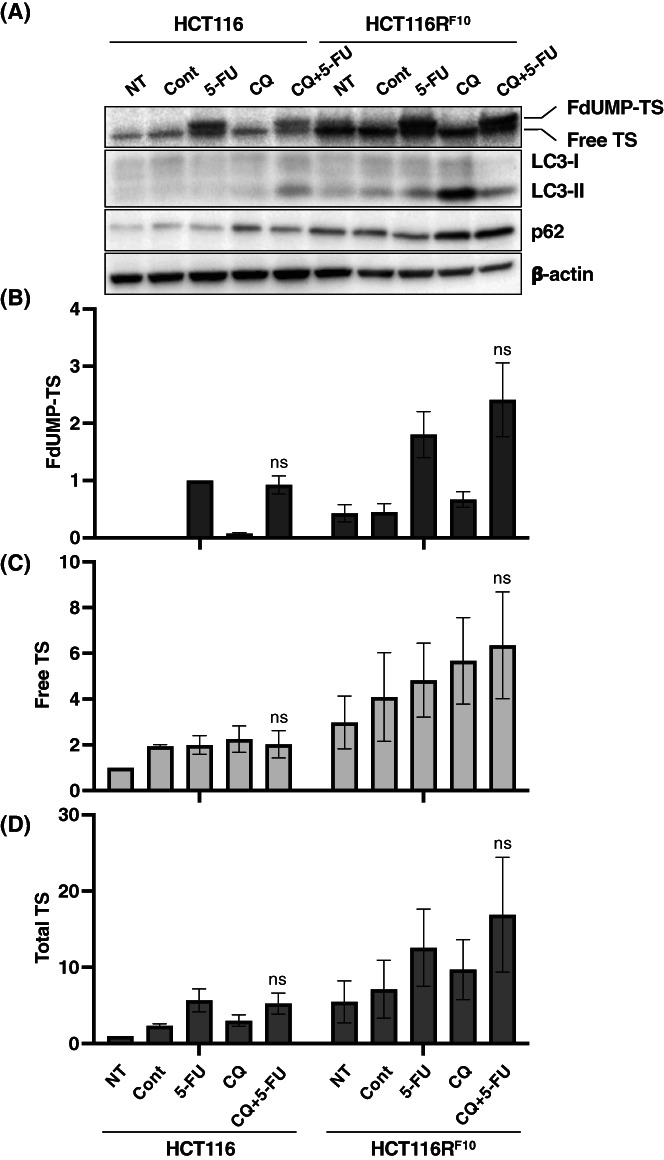
Protein dynamics of two TS forms, FdUMP‐TS and free TS, after treatment with the combination of the autophagy inhibitor chloroquine and 5‐FU in 5‐FU‐resistant HCT116R^F10^ cells and parent HCT116 cells. (A) Protein levels of FdUMP‐TS, free TS, LC3, p62, and beta‐Actin after treatment with the combination of chloroquine (CQ) and 5‐FU in HCT116R^F10^ cells and HCT116 cells. At 24 h after co‐treatment with CQ and 5‐FU, cells were harvested and lysed; then, whole cell lysates were prepared. The protein patterns of LC3, namely LC3‐I and LC3‐II, and p62 were used as autophagic markers. The protein levels of beta‐Actin were used as an internal control. Data are representative of three independent experiments. NT, non‐treated group (no drug and no solvent). Cont, control (solvent alone). 5‐FU, treatment with 100 μM 5‐FU. CQ, treatment with 10 μM CQ. CQ + 5‐FU, combination of 10 μM CQ and 100 μM 5‐FU. (B) FdUMP‐TS protein, (C) free‐TS protein, and (D) total TS protein levels in HCT116R^F10^ cells and HCT116 cells. Levels of FdUMP‐TS are indicated by the ratio of the FdUMP‐TS density to beta‐Actin density in each experimental group to that of parental HCT116 cells at the 5‐FU‐treated stage. Levels of free TS or total TS are indicated as the ratio of the individual TS density to beta‐Actin density for each experimental group relative to the value for the parental HCT116 cells at the untreated stage (NT, non‐treated group). Results are presented as the average of three independent experiments and error bars show the SE of triplicate experiments. Student's *t*‐test, ns indicates not significant (5‐FU vs. CQ plus 5‐FU in each cell line), one‐way ANOVA, *p* < 0.0001 (for FdUMP‐TS levels in all groups), *p* = 0.1176 (for free TS levels in all groups), *p* = 0.1047 (for total TS levels in all groups). [Correction added on [7^th^ December 2022], after first online publication: Figure 4 has been replaced with new figure and the following text has been removed from the caption: “White bar, free‐TS form; gray bar, FdUMP‐TS form”.]

We investigated the hypothesis that the FdUMP‐TS is degraded by autophagy, which results in resistance to 5‐FU. As shown in Figures 4A–D, the FdUMP‐TS, free‐TS, and total TS protein levels were significantly higher in HCT116R^F10^ cells than in HCT116 cells at the untreated stage and after treatment with 100 μM 5‐FU for 24 h. Interestingly, the inhibition of autophagy by CQ increased the accumulation of the FdUMP‐TS in HCT116R^F10^ cells (Figure 4A). The level of FdUMP‐TS was about 2‐fold higher in HCT116R^F10^ cells after combined treatment with CQ and 5‐FU than after treatment with 5‐FU alone; however, no similar difference was observed in HCT116 cells (Figure 4A,B). The patterns of free‐TS and total TS protein levels were similar to those of FdUMP‐TS (Figure 4A,C,D). Furthermore, the levels of microtubule‐associated protein 1A/1B‐light chain 3 (LC3), namely LC3‐I and ‐II, well‐characterized hallmarks of autophagy,^33^ were slightly higher in HCT116R^F10^ cells than in HCT116 cells at non‐treated (NT), control, and 5‐FU treated stages (Figure 4A). Likewise, the intracellular levels of another autophagy marker, Sequestosome 1 (SQSTM1), designated p62, were increased in HCT116R^F10^ cells in non‐treated, control, and 5‐FU treated conditions (Figure 4A). Importantly, intracellular LC3‐II and p62 levels were higher in HCT116R^F10^ cells than in HCT116 cells after treatment with CQ alone or with a combination of CQ and 5‐FU (Figure 4A). These results indicate that autophagy is enhanced in 5‐FU‐resistant HCT116R^F10^ cells compared to that in HCT116 cells and that TS, especially FdUMP‐TS, is eliminated via autophagy in 5‐FU‐resistant CRC cells.

## 4 DISCUSSION

Autophagy is a physiological process that maintains metabolism and cellular homeostasis by recycling damaged cellular components, e.g., abnormal proteins and/or damaged organelles.^26^, ^34^, ^35^ The autophagic machinery is an intracellular degradation system mediated by the fusion of double‐membrane vesicles called autophagosomes, enveloping cytoplasmic components and organelles, to lysosomes to form autolysosomes, which degrade the encased contents.^26^, ^34^, ^35^ In cancer biology, autophagy plays dual roles in tumor promotion and suppression, and contributes to cancer cell development and proliferation.^26^, ^35^, ^36^ Several anticancer drugs, e.g., 5‐FU and cisplatin, can regulate autophagy.^37^, ^38^, ^39^, ^40^ Similarly, several studies have reported that autophagy plays an important role in drug sensitivity and resistance to 5‐FU. The inhibition of autophagy with 3‐MA enhances 5‐FU‐induced apoptosis in mouse colon26 and human HT29 CRC cells.^27^ In addition, the inhibition of autophagy with 3‐MA augments 5‐FU‐induced apoptosis in 5‐FU‐sensitive human HCT116 and DLD‐1 cells and in 5‐FU‐resistant DLD‐1/5‐FU CRC cells.^28^ Another autophagic inhibitor, CQ, potentiates the anticancer effect of 5‐FU in HT29 and colon26 CRC cells.^29^, ^30^ On the other hand, Yao et al. reported that autophagy is downregulated in 5‐FU‐resistant human SNUC5 CRC cells.^41^ Interestingly, Nabeya et al. demonstrated that the lysosomal protease calpain regulates FdUMP‐TS ternary complex levels, associated with chemosensitivity to 5‐FU, in several human gastric cancer cells.^42^ This intriguing finding suggests that calpain may reduce the chemosensitivity of human gastric cancer cells to 5‐FU, possibly by the degradation of the FdUMP‐TS ternary complex. Recently, we reported that the TS enzyme, which is the target of FdUMP, acts as a resistance factor by trapping FdUMP in 5‐FU‐resistant HCT116R^F10^ cells.^25^ Therefore, the trapping of FdUMP by TS may confer resistance to 5‐FU. We also predict that this trapping and the regulation of the accumulation and elimination of the FdUMP‐TS ternary complex are responsible for direct resistance to 5‐FU.

**FIGURE 5:**
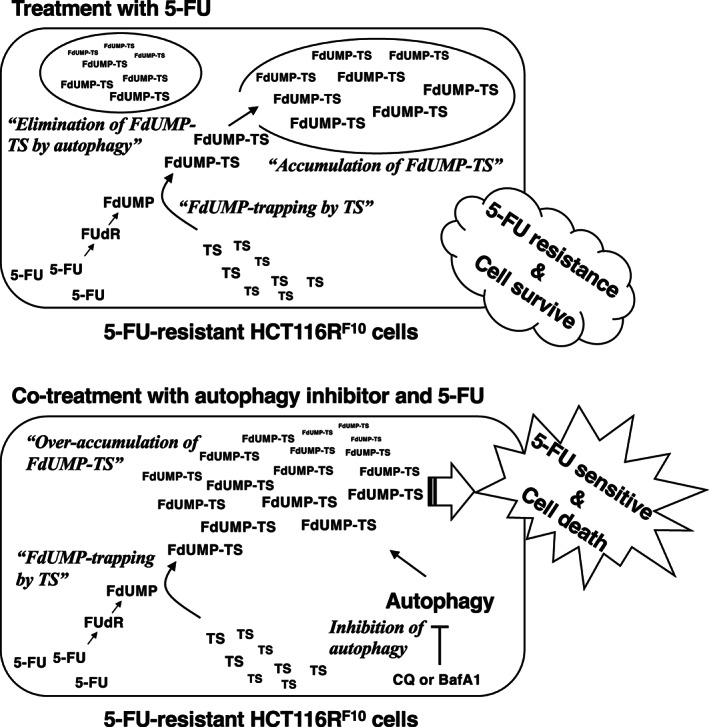
Predictive model of the elimination of the ternary complex thymidylate synthase by autophagy in 5‐FU‐resistant HCT116R^F10^ cells. [Correction added on [7^th^ December 2022], after first online publication: In Figure 5, “5‐FU resistance HCT116 cells” has been corrected to “5‐FU‐resistant HCT116R^F10^ cells”.]

In this study, we found that 5‐FU‐resistant HCT116R^F10^ cells have greater sensitivity to autophagy inhibitors (i.e., CQ and BafA1) than that of parental HCT116 cells. In particular, the combination of 5‐FU and autophagy inhibitors enhanced the anticancer effects of 5‐FU in HCT116R^F10^ cells. Furthermore, the inhibition of autophagy enhanced the accumulation of the FdUMP‐TS in HCT116R^F10^ cells. Our findings suggest that the regulation of FdUMP‐TS ternary complex levels by autophagy contributes to resistance to 5‐FU. Therefore, it is likely that 5‐FU‐resistant HCT116R^F10^ cells gained resistance via alterations in the storage of active free TS, the trapping of FdUMP by TS, and the elimination of the FdUMP‐TS ternary complex by autophagy (Figure 5). Meanwhile, the inhibition of autophagy increased the levels of free TS in HCT116R^F10^ cells. This finding indicates that autophagy in 5‐FU resistant HCT116R^F10^ cells acts as degradation machinery for rapidly and overproduced FdUMP‐TS and various intracellular proteins, including free TS. We assume that FdUMP‐TS is selectivity degraded by the ubiquitin‐proteasome system in parallel with this autophagy machinery. To test this hypothesis, we further investigated the involvement of the ubiquitin‐proteasome system in FdUMP‐TS degradation in the parental HCT116 cells and 5‐FU resistant HCT116R^F10^ cells. Our findings provide a better understanding of the mechanism underlying 5‐FU resistance and may lead to the development of anticancer strategies to improve sensitivity to 5‐FU‐based chemotherapy.

## AUTHOR CONTRIBUTIONS

A.S. conceived and designed the project. N.N., C.K., and A.S. acquired the data. N.N., Y.O., and A.S. analyzed and interpreted the data. N.N. and A.S. wrote the paper. All authors have read and agreed to the published version of the manuscript.

## FUNDING INFORMATION

This research received no external funding.

## CONFLICT OF INTEREST

The authors declare no conflict of interest.

## Supporting information


Figure S1

Figure S1 Legend
