# Supplementary figures and images for: Regulation of 5‐fluorodeoxyuridine monophosphate‐thymidylate synthase ternary complex levels by autophagy confers resistance to 5‐fluorouracil
Source: FASEB Bioadv. 2022 Nov 11;5(1):43–51. doi: 10.1096/fba.2022-00099 (PMC9832531; doi:10.1096/fba.2022-00099)

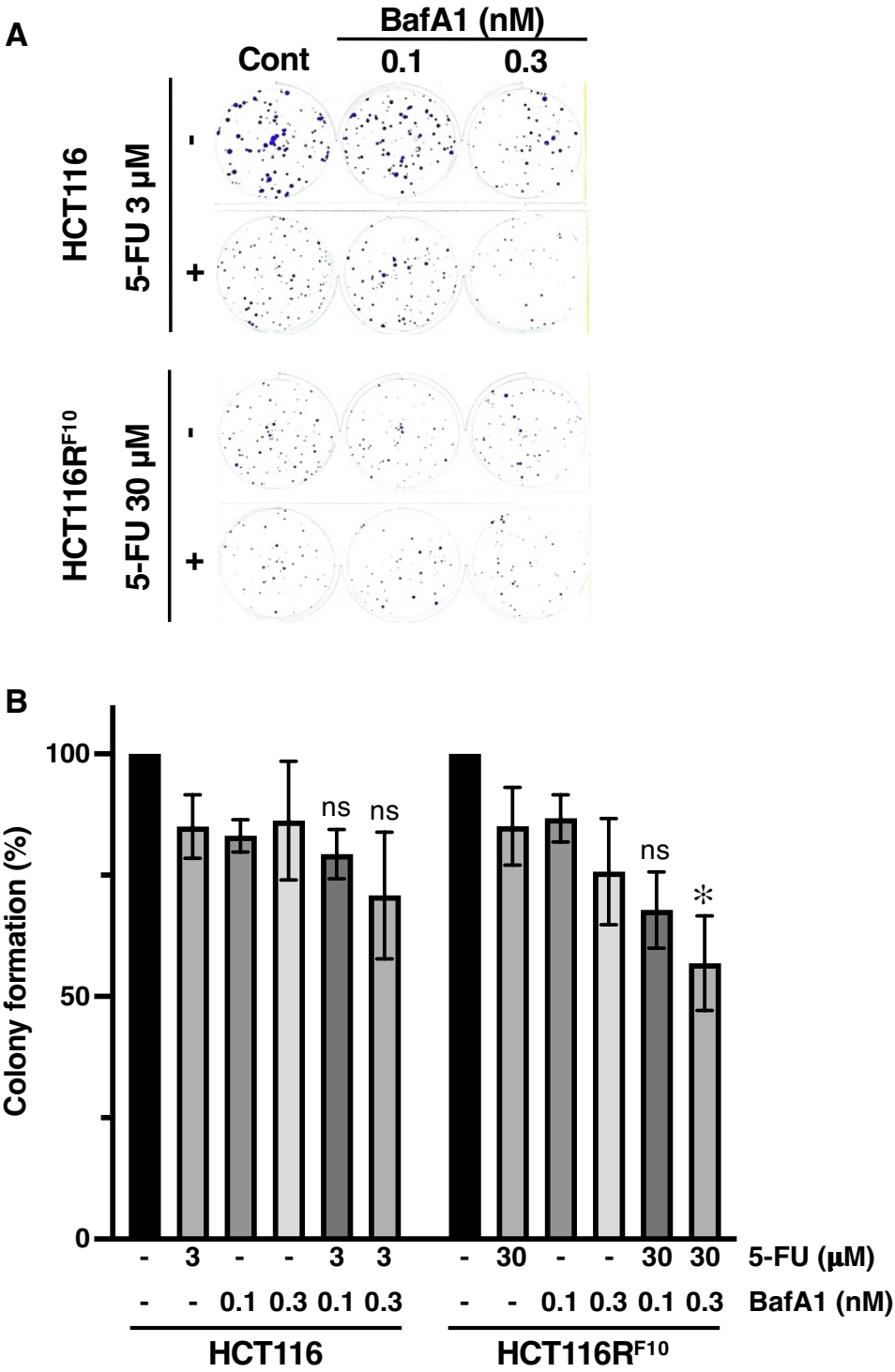

Supplement: Supplementary file 1 — Figure S1 [file FBA2-5-43-s002.pdf]
