# Supplementary material for: Regulation of 5‐fluorodeoxyuridine monophosphate‐thymidylate synthase ternary complex levels by autophagy confers resistance to 5‐fluorouracil
Source: FASEB Bioadv. 2022 Nov 11;5(1):43–51. doi: 10.1096/fba.2022-00099 (PMC9832531; doi:10.1096/fba.2022-00099)
Supplement: Supplementary file 2 — Figure S1 Legend [file FBA2-5-43-s001.docx]

**Supplementary Figure 1. Anticancer effect of the combination of 5-FU and the autophagy inhibitor bafilomycin A1 in 5-FU-resistant HCT116R^F10^ cells and parent HCT116 cells.** (A) Image of colony formation in HCT116R^F10^ cells and HCT116 cells after co-treatment with bafilomycin A1 (BafA1) and 5-FU. (B) Anticancer effect of the combination of BafA1 and 5-FU in HCT116R^F10^ cells and HCT116 cells determined by a colony formation assay. Cells were treated with the indicated concentrations of BafA1 and 5-FU and incubated for 10 days. Colony formation (%) is presented as the average of three independent experiments, with error bars showing the SE of triplicates. Student’s *t*-test (5-FU *vs.* BafA1 plus 5-FU in each cell line), ns indicates not significant, **p* < 0.05, one-way ANOVA, *p* = 0.0326.
